# The Mechanical Performance of Recycled Slate Waste Fiber Composites Based on Unsaturated Polyester Resins

**DOI:** 10.3390/ma16176041

**Published:** 2023-09-02

**Authors:** Rocío Ruiz-Bustos, Antonio López-Uceda, María Isabel López-Martínez, Joost Van Duijn

**Affiliations:** Department of Mechanics, School of Engineering Sciences, Campus Universitario de Rabanales, Edificio Leonardo da Vinci, Ctra. Madrid-Cádiz Km. 396, 14071 Córdoba, Spain; p62louca@uco.es (A.L.-U.); q12lomam@uco.es (M.I.L.-M.); me2vavaj@uco.es (J.V.D.)

**Keywords:** fibrous composites, mechanical properties, recycled slate fibers, unsaturated polyester matrix

## Abstract

In the last few decades, there has been increasing social awareness for environmental conservation, which is driving the development of composite materials based on natural fibers. These new materials have interesting properties that allow for their use in a variety of applications. This study deals with the development of composite materials based on unsaturated polyester resins reinforced with recycled mineral fibers, such as slate fibers obtained from slate production waste, which have similar properties to glass fiber. The mechanical properties of these composites have been determined by tensile and flexural/bending tests. The influence of various variables such as matrix composition (flexible polyester content) and the weight percentage of fiber added to mechanical properties were evaluated. The flexible/rigid polyester content varied from 0 to 40% and the fiber one from 0 to 30 wt%. Composites with ≥20 wt% of slate fiber reinforcement are shown to have tensile (35 MPa) and flexural (57 MPa) strengths that can compete with materials reinforced with artificial fibers.

## 1. Introduction

In the last few years, the use of composite materials has seen rapid growth, gradually replacing certain traditional materials in some cases. Most of these commercial composite materials are, however, reinforced with artificial fibers such as fiberglass, carbon and aramid.

In the last decade, due to growing concern for the environment, the search for and development of new materials that do not harm the environment has increased. Consequently, in the field of composite materials, the use of reinforcements with natural fibers instead of those traditionally used is being studied. Vegetable fibers such as flax, sisal or hemp have already been used to manufacture products with reduced environmental impact [1]. Previous studies on the manufacturing of natural fiber-based composites have concentrated on those with vegetable origins. In particular, Rodríguez [2] focused on the use of banana pseudo stem fibers (FSP) as reinforcement of composite materials with a polyester matrix. Materials with treated and untreated banana fibers were manufactured using the manual molding technique. Mechanical resistance tests showed that the polyester compounds with modified FSP had higher mechanical resistance than the composite materials with untreated banana fibers. Apart from natural fibers, synthetic fibers have been studied in diverse matrixes. Jiang et al. [3] researched the use of polypropylene fiber, along with nano clay on lime soil. Zhang et al. [4] investigated the effect of polyvinyl fiber on geopolymer mortars. Whang et al. [5] studied the effect of polyethylene fiber on the performance of cementitious composites. Owen et al. [6] used recycled nylon fibers and other industrial plastic wastes for producing floor-paving tiles. Jhang et al. [7] made composite nonwoven fabrics by incorporating different polyester fibers with high resilience and a low melting point.

Research carried out by Akil et al. [8] developed polypropylene matrix composite materials reinforced with natural fibers (sisal, kenaf, hemp, jute and coconut fiber) that were manufactured via compression molding using a layer-stacking method. All materials were processed with a 40% weight of reinforcing fiber, except for kenaf-reinforced composites, which were also made with 30% and 50% fiber weight, to investigate the effect of fiber content on the mechanical properties of composite materials. These materials were tested, and new composites were compared with the different natural fibers used as reinforcement. In addition, these properties were also compared with those of composite materials with a polypropylene matrix and fiberglass reinforcement. The kenaf-reinforced, hemp-reinforced and sisal-reinforced composite materials had similar tensile strengths and moduli, but in impact tests, the hemp properties outperformed those of kenaf. The tensile modulus, impact strength and tensile strength of the kenaf-reinforced composites increased with the percent of the fiber weight. Coir-reinforced composites showed the lowest mechanical properties, but their impact resistance was higher than jute- or kenaf-reinforced composites. The specific properties of composites reinforced with natural fibers were, in some cases, better than those of composite materials reinforced with fiberglass. Fajrin et al. [9] found that similar results could be obtained when using Kenaf fiber waste. The use of kenaf fiber with a different orientation in an unsaturated polyester resin was also studied, achieving the best results for a longitudinal arrangement. These composites could be a feasible material for a broad variety of construction applications. This suggests that natural fibers have the potential to replace glass fibers in many applications that do not require a very high load [10].

Bhoopathi et al. [11] carried out a study in which hybrid composite materials of three different types were made. These had an epoxy matrix and different fibers as reinforcement. The first one was made with an epoxy matrix reinforced with banana and glass fibers, another type was reinforced with hemp and glass fibers, and finally, a laminate was obtained with an epoxy matrix and glass fiber reinforcement of hemp and banana. The manufacturing process of the three types of composite materials was carried out via hand molding and the mechanical characterization was carried out with tensile, bending and impact tests. The hybrid material that presented the best properties was reinforced with fiberglass, banana and hemp. Due to its properties, this material could be used as an alternative material to composite materials reinforced with synthetic fibers.

Recently, the use of natural fibers of mineral origin, such as basalt fibers [12,13] or slate obtained through slate waste, has begun to be investigated. The latter could be of great interest as an alternative to glass or carbon, since they have similar characteristics. However, these new fibers still need a great deal of study and development so that all their characteristics, advantages and disadvantages may be known in order for manufacturers to incorporate them in their products. Slate waste comes from cutting and manufacturing when it is processed for roofing or flooring purposes, for instance. It is becoming a problem difficult to deal with for slate producers [14,15].

Regarding basalt and slate fibers, in recent years, basalt has been increasingly studied as a candidate for the replacement of glass in composite materials. However, slate fiber has been less investigated for the manufacture of composite materials [12].

Research carried out by Samper et al. [16] focused on the development of new laminated composite materials called ‘green composites’, which were based on the use of epoxidized vegetable oil (ELO) as matrix and reinforcement slate fiber fabrics (obtained from slate waste) with different treatments and with coupling agents such as silanes or titanates. ELO matrix and slate fiber fabric composite laminates were fabricated using a resin transfer molding (RTM) system. A vacuum pump was connected to the ventilation hole of the mold in order to improve the evacuation of air and avoid the entrapment of bubbles in the laminate. For the realization of each composite, four layers of slate fiber fabric were used, positioned in the same orientation. These laminated composites contained 56% slate fiber. The mechanical properties of these laminated composite materials were tested with tensile, flex and impact tests. The effects of different silane coupling agents on fiber-matrix interface phenomena were studied with scanning electron microscopy. Through treatment with coupling agents, fiber-matrix interaction was improved to promote a coupling effect between both components; this coupling effect improved the charge transfer from the matrix to the fiber, which led to a better mechanical performance. A ‘green composite’ with very good mechanical properties was obtained, in which the slate fibers were modified with the coupling agents glycidyl silane and titanate, and epoxidized linseed oil was used as the matrix. This presented a flexural strength of 402.1 MPa and a flexural modulus of 19.7 GPa; the tensile strength was 359.1 MPa and the Young’s Modulus was 25.6 GPa. The good resistant properties it presented allowed it to replace traditional composites made with conventional fiberglass and basalt-reinforced laminates. In addition to working with the slate fiber fabric reinforcement, basalt fabric was also used, and both cases, the fibers were mixed with vegetable oil matrices.

Other research carried out by Samper [17] focused on the use of short slate fiber obtained from slate waste that was treated with different coupling agents as a reinforcement material for thermoplastic polymers, specifically, for high-density polyethylene obtained from sugar cane. The effect of the different coupling systems and the slate fiber content were evaluated by scanning electron microscopy (SEM), dynamic thermomechanical analysis (DTMA) and the mechanical characteristics from tensile, bending and impact tests. Here, composite materials were manufactured with different coupling agents, but all with the same amount of slate fiber (20% weight), to evaluate which was the best compatibilization system between the polyethylene matrix and slate reinforcement. Once the best compatibilization system was selected, composites were manufactured with different amounts of slate fiber as reinforcements, with ranges varying between 5% and 30% in weight to evaluate the effect that the amount of reinforcement had on the composite material used. The manufacturing process of composite materials was carried out via extrusion and, later, injection molding. The results showed that the use of silane coupling agents leads to better fiber-matrix interaction, which has a positive effect on mechanical properties overall. Regarding slate fiber content, from a 10% slate fiber weight, mechanical resistance properties increased, and the energy absorbed in the Charpy impact test was higher than in the unreinforced matrix. The most interesting results obtained were for the composite materials containing 30% slate fiber weight previously treated with propyltrimethoxy silane (PTMS), which demonstrated an increase in tensile strength and flexural strength of around 16% and 18%, respectively. The tensile strength and elastic modulus for the unreinforced material had values of 19.6 MPa and 373 MPa, respectively. For materials reinforced with a 30% slate fiber weight, tensile strength was 22.7 MPa, and the elastic modulus was 2150 MPa. Regarding flexural tests, similar improvements were also obtained. The flexural strength and flexural modulus for the unreinforced material had values of 23 MPa and 805 MPa, respectively. For materials reinforced with a 30% slate fiber weight, flexural strength was 30 MPa and the elastic modulus was 2864 MPa [18].

Also, other authors such as De Carvalho et al. [19] developed polypropylene-based composite materials by using different percentage charges of slate dust. In this case, slate fibers did not come from waste, but directly from waste slate dust that was obtained from cutting and handling slate rock. The manufacture of the composites was carried out in a mixer where the slate powder and the polypropylene were mixed in different proportions; maleic anhydride was also included in the mixture to induce the chemical modification of the polypropylene in order to improve the interactions between the polymer and slate dust. The samples were characterized through infrared spectroscopy and scanning electron microscopy, and it was found that the chemical modification of polypropylene with maleic anhydride gave rise to a partial improvement of the interactions between the slate powder and the polymer. Materials containing 5 and 10% slate produced samples with similar optical characteristics to the original slate rock. Mechanical properties were evaluated via tensile tests, with which it was shown that the slate particles did not significantly alter the mechanical resistance of polypropylene. Therefore, the incorporation of slate powder into the polypropylene matrix appears to be a potential technology for the production of systems with some mechanical properties comparable to those of pure polypropylene at low cost, optical properties similar to those of pure slate and the ability to minimize environmental problems derived from the presence of waste produced in industrial activities. In a related work, Kahn et al. [20] developed slate waste powder poly- (lactic acid) (PLA) based composite for 3D printer filaments. Their study shows that, apart from influencing mechanical properties, the addition of slate waste powder significantly affects the composites’ dynamic mechanical properties, such as storage, loss modulus and the damping factor. They conclude that the optimum composite composition for printing applications is one containing a 10% slate powder weight. Other uses of slate waste have been as aggregate in the production of mortars [21] and as filter sand for the treatment of drinking water [22]. While the work mentioned above focused on the use of slate particles or fiber fabrics as reinforcement in polymers, no published reports have been found on their use in the manufacture of thermosetting polymer matrix composite materials. In this work, the study of an unsaturated polyester resin matrix, a material that is widely used in construction, as a matrix of composite materials reinforced with randomly oriented slate fibers is reported. For this purpose, mechanical properties have been performed on composites made with different proportions of rigid/flexible polyester resin and different amounts of slate fiber.

## 2. Materials and Methods

### 2.1. Preparation of the Testing Samples

**Figure 1:**
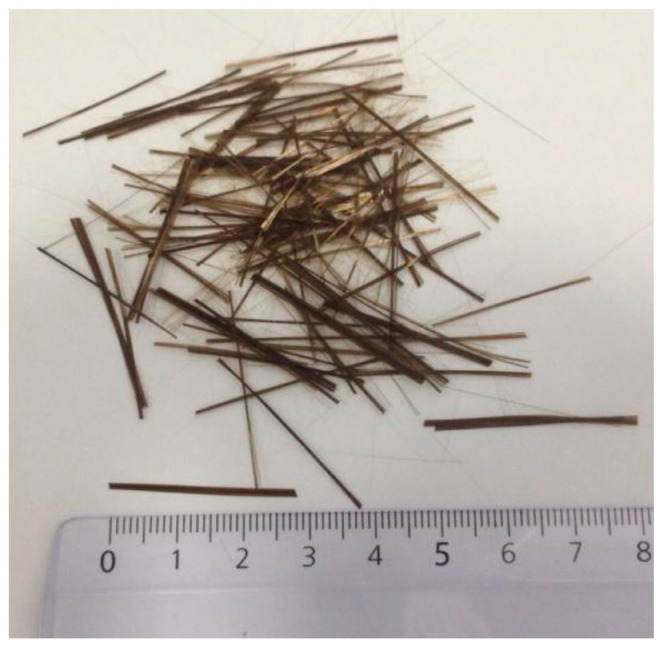
Sample of recycled slate fibers used to make the composites used in this study. The fibers are about 30 mm long and have an average diameter of 15 to 23 µm.

The matrix of the composite materials was prepared using two types of unsaturated polyester resins supplied by Plastiform (Plastiform, Madrid, Spain). One of them is orthophthalic unsaturated polyester resin (rigid polyester) whose trade name is Activated Cronolita 1012 [23]. It is a general purpose pre-accelerated resin primarily used for fiberglass laminates and does not include a catalyst. The other resin used to manufacture the matrix is flexible or flexibilizing unsaturated polyester whose trade name is Activated flexibilizing Chronolite 3015 [24], which also does not include the catalyst. The catalyst used with the two resins described above was also supplied by Grupo Plastiform; it is a 50% methyl ethyl ketone peroxide, its trade name is Catalyst C-201 [25], and it is specific to pre-accelerated polyester resins. Its dosage is a weight of 1.5% resin. The reinforcing material used for the composites was bright brown recycled slate fiber (Figure 1), which is about 30 mm long with an average diameter between 15 and 23 µm and is supplied by the company (Mifribra, Orense, Spain). For the manufacture of the silicone molds in which the composites have been manufactured, silicone and catalyst have been used, both supplied by Grupo Plastiform. The silicone used is a polycondensation silicone for making molds for resin castings, which does not include the catalyst (Silicone RTV 3325). The catalyst used, known as SPE Catalyst, is one specially for polyester resin castings and is compatible with RTV 3325 Silicone. Its dosage was a weight of 5% silicone.

The manufacture of composite samples reinforced with slate fibers was carried out using a manual molding system with an open mold—three specimens for each of the tests conducted. In the elaboration of the composite materials, different percentages of two types of unsaturated polyester resins were used to constitute the matrix, and different percentages of slate fiber were used as reinforcement (Table 1). Three types of composites were developed depending on the following compositions of their matrix: a matrix made exclusively of rigid polyester, a matrix made up of a weight of 80% rigid polyester and 20% flexible polyester, and a matrix of a weight of 60% rigid polyester and 40% flexible polyester. Each type of matrix was reinforced with different loads of slate: 5, 10, 20 and 30% in fiber weight. Unreinforced specimens were also prepared for the purpose of obtaining the properties of the resins without reinforcement to study how different amounts of fiber reinforcement influence mechanical properties. The fibers were introduced into the mold with the mixture of resins and catalyst, using the help of forceps so that they could all be impregnated in the matrix. After finishing the fiber addition process, the specimen was left in the mold to finish curing at room temperature (20.5 °C), which took approximately 4 h. After 24 h, the specimens were removed from the mold, labeled with their corresponding designation, which was based on the composition of the matrix and the amount of reinforcement, and placed in the oven for 8 h at a temperature of 40 °C to finalize the curing. In all samples a weight of 1.5% catalyst was added.

### 2.2. Mechanical Testing

#### 2.2.1. Tensile Tests

**Figure 2:**
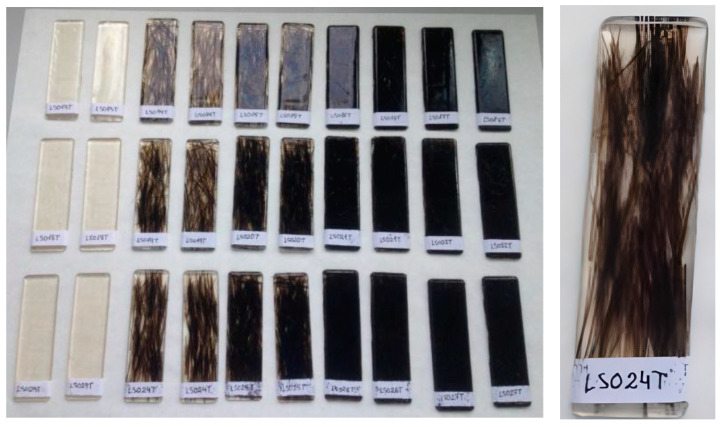

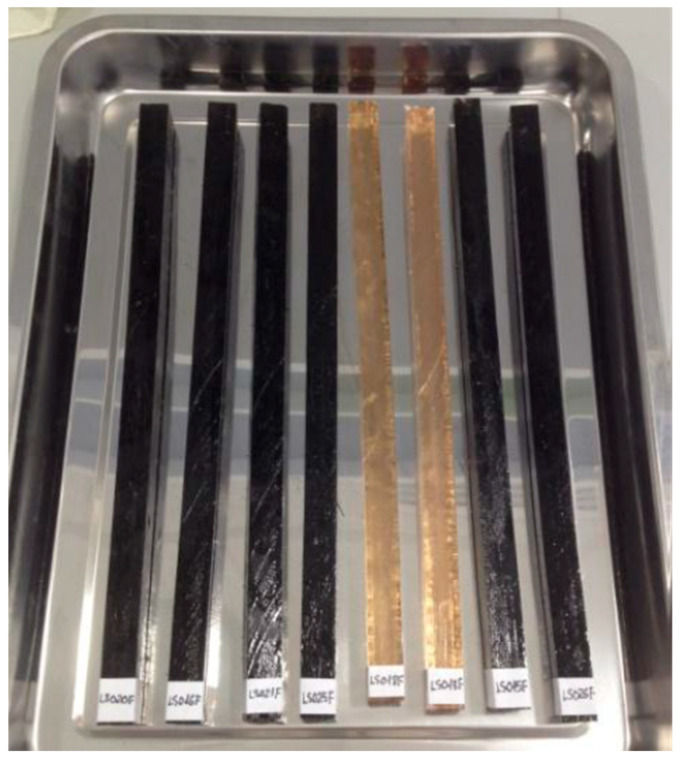
(**Top**) Samples used for the tensile strength tests (with increasing fiber content going from left to right and increasing flexible polyester content going from **top** to **bottom**) and on the right, a sample (n°12) showcasing the orientation of the fiber. (**Bottom**) Examples of the samples used for the flexural strength test.

Following the same manufacturing procedure previously developed, specimens for the tensile test were designed (Figure 2), which had the following dimensions: 110 × 31 × 3 mm.

**Figure 3:**
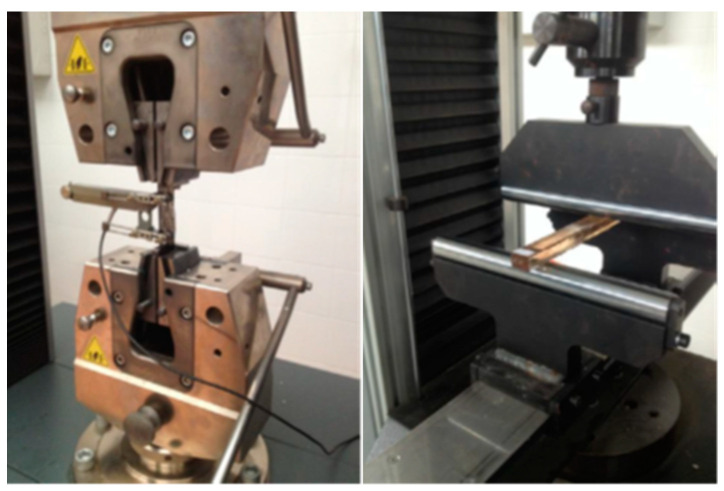
Examples of the tensile test (**left**) and flexural test (**right**) setups used in this study.

The machine used to carry out the tensile tests was the ZWICK Z100 Electromechanical Universal Machine for static tests and was controlled through TestExpert Software Version II. The tensile tests were carried out under the UNE-EN ISO 527 standard [26] (Plastics—Determination of tensile properties) with a loading speed of 2 mm/min at room temperature. A clip-on extensometer with a calibrated distance of 50 mm was used for measuring strains, instead of the crosshead displacement of a testing machine (Figure 3).

A modulus of elasticity in traction was calculated using the Equation (1):

E = ∆σ/∆ε,(1)

where Et is the modulus of elasticity in traction (MPa) and ∆σ/∆ε is the slope of the stress (in MPa) versus ε linear regression between the ε = 0.05% and ε = 0.25%.

Using the extensometer, the strain was evaluated using Equation (2):

ε = ∆Lo/Lo,(2)

where ε is the value of the uniaxial strain expressed as a dimensionless unit, Lo is the reference length of the test specimen (mm) and ∆Lo is the increment in the length of the specimen measured (mm).

Maximum stress values were calculated following Equation (3):

σ_M_ = F_max_/A, (3)

σ_M_ is the maximum stress value, expressed in megapascals (MPa); 

F_max_ is the maximum measured force, expressed in newtons (N); 

A is the initial cross-sectional area of the specimen, expressed in square millimeters (mm^2^). 

The toughness in tensile strength was calculated via integration of the load-displacement curve from zero to the maximum load reached.

#### 2.2.2. Flexural Tests

For the bending test, the manufactured specimens have dimensions of 340 × 25 × 17 mm.

The machine used to carry out the tests was the IBERTEST Electromechanical Materials Testing Machine-EUROTEST 100 Series. The bending tests were carried out under the UNE-EN ISO 14125 standard [27] (Fiber-reinforced plastic compounds—Determination of bending properties). According to the three-point bending procedure, the specimens were tested under a span of 280 mm, and a constant rate of 1 mm/min was imposed at the mid-span of the specimen and recorded until fracture.

Flexural strength values were calculated following Equation (4):

σ = 3FL/2bh^2^, (4)

where

σ is the maximum stress value in question, expressed in megapascals (MPa); 

F is the maximum measured force concerned, expressed in newtons (N); 

L is the span of the specimen, expressed in millimetre (mm); 

b is the width of the specimen, expressed in millimetre (mm); 

h is the thickness of the specimen, expressed in millimetre (mm).

## 3. Results and Discussion

Tensile tests were carried out for three series of samples: the first with a rigid polyester matrix and variable amounts of slate fiber (0, 5, 10, 20 and 30% weight); the second with the same amounts of slate fiber, but with 20% flexible polyester added to the continuous phase; and the third, which consisted of a matrix with 40% flexibilizer, and again, the same amounts of fiber. Two test tubes of each type were manufactured to be tested twice in order to verify the results obtained.

**Figure 4:**
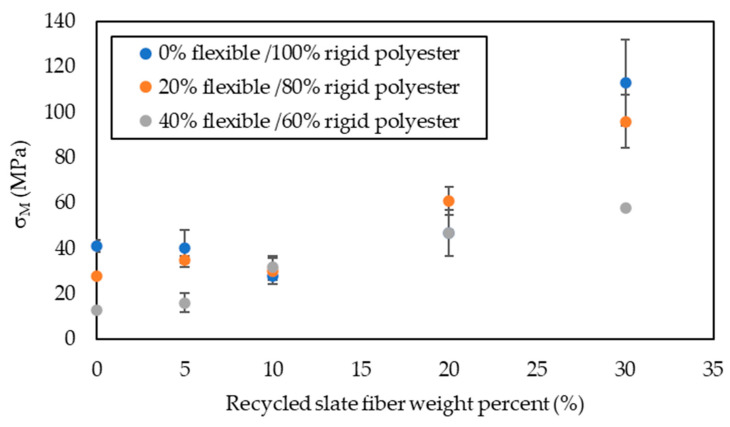
Results of maximum stress in the tensile strength test, σ_M_, for specimens with different percentages of flexibilizer as a function of the amount of slate fiber added.

**Figure 5:**
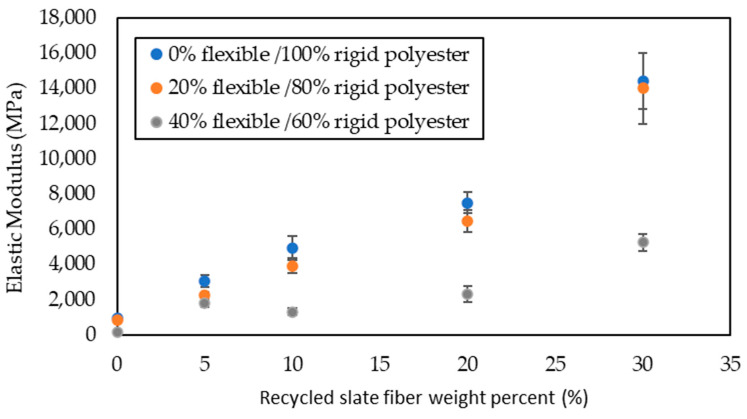
Elastic modules in the tensile strength test with different flexible polyester contents as a function of slate fiber content.

**Figure 6:**
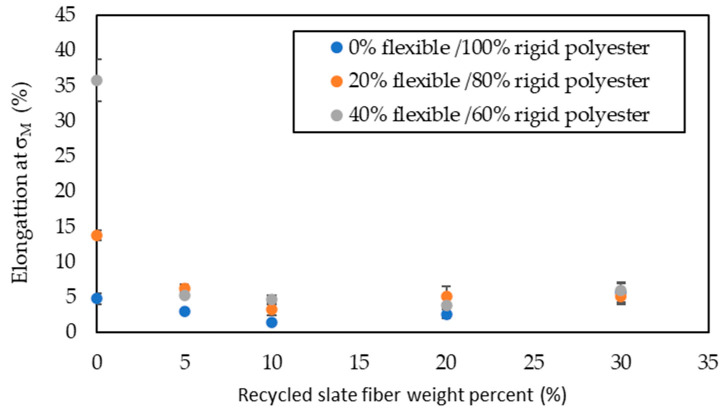
Elongation at σ_M_ in the tensile strength test with different flexible polyester contents as a function of slate fiber content.

**Figure 7:**
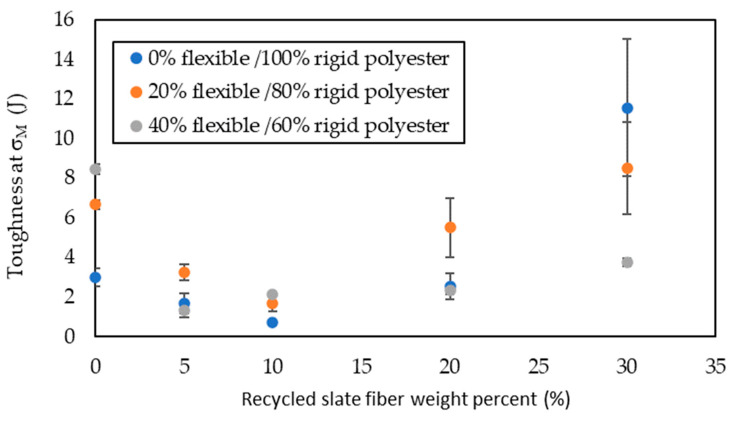
Toughness at σ_M_ in the tensile strength test with different flexible polyester contents as a function of slate fiber content.

The data obtained from the stress versus strain graphs are shown in Figure 4, Figure 5, Figure 6 and Figure 7.

Regarding tensile strength (Table 2), for composite materials whose matrix is formed exclusively by rigid polyester, the increase in σ_M_ was obtained from a 20% weight of reinforcing fiber. With this percentage weight, the value of σ_M_ increased by 18% with respect to the unreinforced matrix. It was with a 30% slate fiber weight that a relevant increase of 183% was achieved with respect to the matrix without reinforcement. Maximum tensile strength was obtained with 30% slate fiber, and its value was 113 MPa. In composite materials whose matrix was made up of an 80% weight of rigid polyester and 20% of flexible polyester (the same as for rigid polyester matrix materials), the increase in tensile strength was obtained from a 20% weight of reinforcing fiber, which can be seen in Figure 2. In this case, the increase in resistance with 20% reinforcement is much more significant, as occurred in Fajrin’s et al. [9] research using kenaf as fiber, rather than that achieved with rigid polyester and the same amount of reinforcement.; the tensile strength value increased by 123%, compared to the unreinforced matrix. Maximum tensile strength reached a value of 96 MPa and was achieved with a 30% in fiber weight, obtaining a 248% increase in strength with respect to the unreinforced matrix. For the third series, whose matrix was made up of a 60% weight in rigid polyester and 40% in flexible polyester, it can be seen in Figure 4 that the tensile strength began to increase from when the minimum amount of reinforcement was used from fiber (5%), but it is from a 10% fiber weight that the tensile strength increased considerably with respect to that of the unreinforced test piece. The highest tensile strength obtained for the test piece was reinforced with a 30% weight in fiber whose value was 59 MPa.

Figure 5 shows the modulus of elasticity calculated from the tensile strength tests for the series described. In the specimens without flexibilizer and in those with 20% of it, there was a clear increase in the elastic modulus as the fiber slate content increased, as occurred in the research of Carbonel et al. [18] and Quiles-Carrilo et al. [28]. The increase was practically linear for the weight percentages of the 20% slate; however, from that point on, the trend was almost exponential. In the samples with 40% flexibilizer, the variation was not linear, but had maximum values for 5 and 20% slate weights. This could be due to the large amount of flexibilizer, which makes mechanical properties depend almost exclusively on the amount of slate. The modulus corresponding to 30% slate fiber and 40% flexible polyester was very low compared to those with a lower flexible polyester content. This was due to the fact that there was hardly any rigid polyester between the slate fibers. Composites with 40% of flexible polyester and 30% of slate fiber presented similar Elastic Modulus values than the equivalent composites of the Sapuan et al. [29] study.

With regards to elongation at maximum tensile strength (Figure 6), there were no relevant differences among the composites with any amount of slate fiber, regardless of flexibilizer incorporation; it ranged between 6.24% and 1.44%. However, in the composite samples with no slate fiber, the elongation at σ_M_ was strongly influenced by the flexibilizer incorporation ratio; when it was 40%, elongation at σ_M_ was near 8 times the one for 0% of flexibilizer and near 3 times in the case of 20% of flexibilizer. Carbonel et al. [12] and Quiles-Carrilo et al. [14] also found that the incorporation of fibers strongly reduced elongation. The toughness at σ_M_ (Figure 7), defined as the energy absorbed until reaching maximum stress during tensile strength tests, experienced a similar trend for each of the series: a decrease as the amount of slate fiber rose to 10% and, after that, an increase up to 30% of slate fiber. For the series with samples of 100% rigid polyester, the increase in toughness with 30% slate fiber was great, reaching a near fourfold increase related to the sample with no slate fiber; for the 20% series, this increase was lower, 28%; and conversely, for the series with 40% flexibilizer, the greatest toughness was for the sample with no slate fiber.

**Figure 8:**
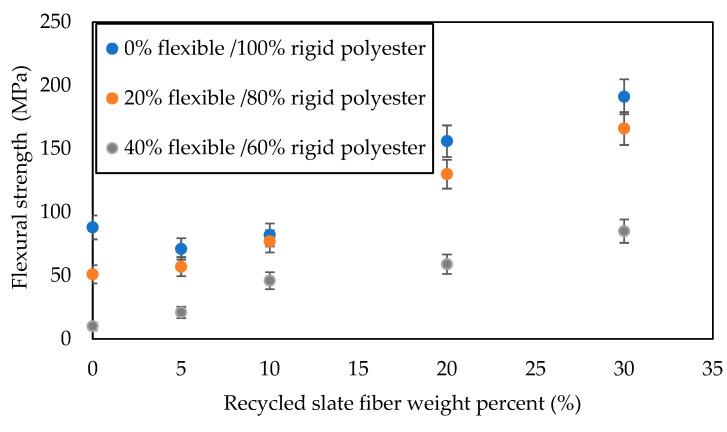
Comparison of the graphs of flexural strength as a function of the % of slate fiber weight for the different series.

On the other hand, from the bending tests (Figure 8, Table 2), it could be deduced that regardless of the composition used to manufacture the matrix, the bending resistance began to increase when reinforcement exceeded 10% in fiber weight. For composites whose matrix was made exclusively from rigid polyester, an increase in flexural resistance was obtained from a 20% slate fiber weight, which is quite significant, as an increase in resistance of 77% was obtained in comparison with the specimen without reinforcement. The highest resistance was obtained for the test tube reinforced with 30% fiber weight, being 191 MPa. Finally, for the specimens whose matrix was made of 60% rigid polyester weight and 40% flexible polyester weight, flexural resistance increased in a more linear way than in the previous cases, obtaining increases in resistance from 5% fiber weight but with much lower results than those described above. The maximum resistance to bending was achieved with the specimen reinforced with 30% fiber weight with a value of 85 MPa. Justification could be found, as in the case of Young’s modulus, that with such high concentrations of slate and flexibilizer, there was not enough rigidity.

When comparing the results obtained in this study with another study carried out by Guermazi et al. [30], it has been verified that composite materials made with slate fiber could present mechanical properties like those of fiberglass-reinforced composite materials but are inferior to composites with carbon fiber reinforcement. Table 3 shows values obtained from other investigations with glass or carbon reinforcements [30]. As the percentages of slate fiber used in this work were lower than those referenced, the value of the magnitudes represented would have to be extrapolated, concluding that, for quantities of slate fiber of approximately 57% or 49% (in comparison with materials with fiberglass and carbon, respectively), higher values of tensile strength and flexural strength would be obtained than in materials with fiberglass reinforcement. However, these would not reach those obtained for equivalent percentages of carbon fiber. Therefore, slate fiber could compete with fiberglass with the advantages of being lighter than fiberglass, having better behavior against alkaline and saline chemical attack, having better resistance to temperature (it has a high tensile strength of up to 500 °C), containing a higher modulus of elasticity and, mainly, being a type of fiber that is obtained from existing slate residues in Spain and therefore contributes to the circular economy. Sapuan et al. [29], who studied the performance of basalt and glass fiber reinforcement in unsaturated polyester–resin hybrid composites, found that basalt presented even better mechanical properties than glass fiber.

**Figure 9:**
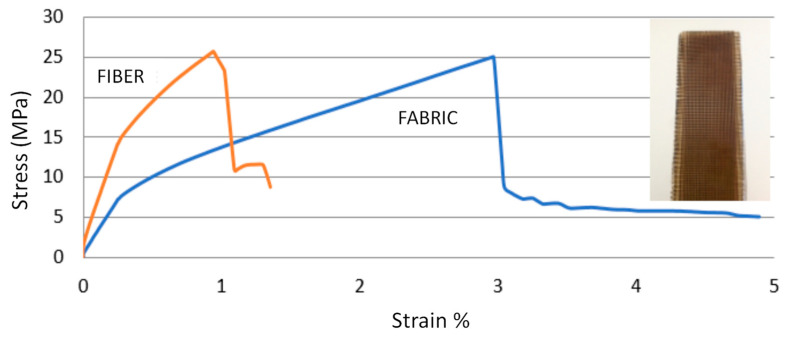
Comparison of the stress-strain graphs for reinforcements with fibers and with mesh (test specimen tested with mesh reinforcement is observed in detail).

On the other hand, in order to determine the influence of the type of reinforcement (fibers, particles or mesh) on mechanical properties, a test specimen was prepared with 10% reinforcement type mesh weight (corresponding to 2 layers of fabric). The manufacturing process for the specimen matrix was the same as for the slate fiber-reinforced specimens (see detail Figure 9) with a 100% rigid polyester matrix. Reinforcement in the shape of a mesh gave the specimen greater deformation than that obtained with fiber reinforcement. Tensile strength remained similar, regardless of whether the reinforcement was fiber or woven, while deformation was affected by changing the shape of the reinforcement. This can be seen in Figure 9 from which it can be observed that for the specimen reinforced with 10% slate fiber, the values of tensile strength and deformation were 26 MPa and 1%, respectively, while for the specimen reinforced with 10% slate fiber fabric, values of 25 MPa were obtained for tensile strength and 3% for deformation. This would indicate that, by keeping the tensile strength practically the same, but increasing the strain, the area enclosed under the curve represented in the Stress-Strain graph was greater. Thus, upper toughness in the material was reinforced with fabric. This shows that by controlling the slate fiber shape, one can design composites with well-defined mechanical properties.

## 4. Conclusions

In this work, through the manufacture of numerous types of composite materials, the influence of the amount of reinforcing fiber on the properties of a thermosetting polymer matrix composite material with different rigid/flexible polyester ratios was evaluated. In addition to the percentage of slate fiber weight to reinforce the material, the influence of different compositions for the matrix on mechanical properties was evaluated. By varying the amounts of flexibilizing polyester, materials with different characteristics were obtained, the general trend being that composites with the greatest amount of flexibilizing agents (40%) and minimal amount of fiber content (5%) have the lowest tensile strength (16 MPa), while those with the maximum amount (30%) have the greatest deformation in tensile strength (6.0%). At the other extreme are materials whose matrix is made up exclusively of rigid polyester that have the highest tensile strength (30% slate fiber, 113 MPa) but the least tensile strain (10% slate fiber, 1.4%). Regarding the amount of slate fiber reinforcement, those reinforced with a 30% weight present the greatest tensile (59–113 MPa) and flexural resistance (85–191 MPa). It can therefore be concluded that there is an optimal amount of flexibilizer to achieve high mechanical resistance and allow some deformation (<40% flexible polyester content), avoiding a brittle fracture of the material, allowing one to obtain materials that can absorb a lot of energy before breaking. Changing from a fiber to a 2-dimensional mesh reinforcement results in an increase in tensile strain (~×3) while maintaining tensile strength. It will therefore be interesting to devise a standardized preparation method for these types of composites.

This would be of great interest, since these composites are beginning to be used for structural rehabilitation, such as the manufacture of flooring, kitchen countertops, false ceilings (removable), blinds and decorative items. In addition to applications in the construction sector, these materials could have a place in the manufacture of sporting goods such as baseball bats, tennis rackets, skis, surfboards and some parts that make up golf clubs thus contributing to the sustainability in the construction sector, while at the same time giving added value to slate production waste.

While the work presented here focuses on mechanical properties at room temperature, it will be interesting to extend this study to other than ambient conditions, as well as the dynamical, mechanical and thermal properties.

## Figures and Tables

**Table 1:** Summary of the samples produced and their composition.

N° Sample	% Recycled Slate Fiber	% Rigid Unsaturated Polyester Resins	% Flexible Unsaturated Polyester Resins
1	0	100	0
2	5	100	0
3	10	100	0
4	20	100	0
5	30	100	0
6	0	80	20
7	5	80	20
8	10	80	20
9	20	80	20
10	30	80	20
11	0	60	40
12	5	60	40
13	10	60	40
14	20	60	40
15	30	60	40

**Table 2:** Summary of the results obtained.

	Tensile Strength								Flexural Strength	
	σ_M_		Elastic Modulus		Elongation at σ_M_		Toughness at σ_M_		σ_M_	
N° Sample	(MPa)		(MPa)		(%)		(J)		(MPa)	
1	41	(3)	930	(150)	4.8	(0.8)	3.0	(0.4)	88	(9)
2	40	(8)	3040	(350)	3.1	(0.1)	1.7	(0.5)	71	(8)
3	28	(2)	4910	(678)	1.4	(0.2)	0.7	(0.1)	82	(9)
4	47	(10)	7490	(598)	2.7	(0.6)	2.5	(0.7)	156	(12)
5	113	(19)	14,400	(1598)	5.7	(1.4)	11.6	(3.4)	191	(14)
6	28	(0)	818	(124)	13.8	(0.7)	6.7	(0.2)	51	(7)
7	35	(2)	2230	(189)	6.2	(0.6)	3.3	(0.4)	57	(8)
8	30	(6)	3930	(415)	3.2	(0.8)	1.7	(0.4)	77	(99)
9	61	(6)	6460	(598)	5.2	(1.4)	5.5	(1.5)	130	(11)
10	96	(12)	14,000	(2015)	5.1	(1.0)	8.5	(2.3)	166	(13)
11	13	(0)	141	(35)	35.8	(3.0)	8.5	(0.3)	10	(3)
12	16	(4)	1800	(254)	5.3	(0.1)	1.4	(0.4)	21	(5)
13	32	(5)	1310	(203)	4.8	(0.5)	2.1	(0.0)	46	(7)
14	47	(1)	2320	(453)	3.8	(0.4)	2.4	(0.2)	59	(8)
15	59	(0)	5240	(466)	6.0	(1.1)	3.8	(0.2)	85	(9)

Standard deviation in brackets.

**Table 3:** Comparison of composites reinforced with glass and carbon fibers [30], with composites with slate fibers used for this study.

Matrix	Reinforcement	% Fiber Weight	σ_Traction_ (MPa)	σ_Flexion_ (MPa)
Epoxy	Glass	48–66	186	241
Epoxy	Carbon	49	242–312	631
Polyester	Slate	30	113	190
